# Fitness of natural willow hybrids in a pioneer mosaic hybrid zone

**DOI:** 10.1002/ece3.2470

**Published:** 2016-10-05

**Authors:** Susanne Gramlich, Elvira Hörandl

**Affiliations:** ^1^ Department of Systematics, Biodiversity and Evolution of Plants (with Herbarium) Georg August University Göttingen Göttingen Germany

**Keywords:** fruit set, germination, *Salix*, seed set

## Abstract

Hybrid fitness is an important parameter to predict the evolutionary consequences of a hybridization event and to characterize hybrid zones. We studied fitness parameters of F_1_ and later‐generation hybrids between the lowland species *Salix purpurea* and the alpine *S. helvetica* that have recently emerged during colonization of an alpine glacier forefield. Fruit production (number of capsules per catkin and fruit set) did not differ between hybrids and parents, but the number of seeds per capsule of F_1_ hybrids was slightly lower than that of later‐generation hybrids and of the parents. Germination rates and seedling growth were tested on three substrates (pH 4.5, 7.0, and 8.0). Germination rates of seeds collected from F_1_ hybrids were lower on acid and neutral substrates, but equal at pH 8.0 compared to all other groups, while the seeds from later‐generation hybrids performed as well as the parents on all three substrates. In seedling growth, the colonizer *S. purpurea* performed better than all other taxa on all three substrates, while hybrids resembled the subalpine species *S. helvetica*. Results suggest that endogenous selection acts against F_1_ hybrids, but favors fitter genotypes in later‐generation hybrids. Exogenous selection via soil pH appears to be weak during seedling establishment. The pioneer vegetation on the glacier forefield may offer sufficient niche space for hybrid seedlings. Owing to the relatively high fitness of the hybrids and the scattered distribution of hybrids and parental individuals on the glacier forefield, this hybrid zone can be assigned to a mosaic model, probably facilitating gene flow and introgression between the parental species. As establishment of the hybrid zone appears to be linked to a colonization process, we propose to call it a pioneer mosaic hybrid zone.

## 1 Introduction

Hybridization is a relatively frequent phenomenon in flowering plants (Arnold, 1997). Nevertheless, the evolutionary role of hybrid formation is still controversial and in details poorly explored (Mallet, 2005). Hybridization can be seen as a breakdown of isolating mechanisms of species, which is maladaptive because of low fertility of hybrids (Mallet, 2005). Other authors emphasize the evolutionary potential of hybridization by increasing variation and by creating new combinations of adaptive traits in the offspring (Rieseberg & Willis, 2007).

Estimation of hybrid fitness is an important factor in predicting the evolutionary consequences of a hybridization event (Arnold, 1997). The evolutionary role of hybridization would be minor if hybrids were consistently less fit than their parental species (Rieseberg & Carney, 1998). In this case, hybridization would not threaten the integrity of the parental species and the probability of introgression of genes would be low (Milne, Terzioglu, & Abbott, 2003; Nagano, Hirao, & Itino, 2015; Zha, Milne, & Sun, 2010). Conversely, if hybrids demonstrated an elevated fitness compared to both parental species, it would be likely that species merge as a result of hybridization and introgression (Arnold, 1992; Rieseberg, 1997; Rieseberg & Carney, 1998). However, the assumptions of total hybrid superiority or total hybrid inferiority are of course the extremes of a continuum (Arnold, Ballerini, & Brothers, 2012). Fitness of the early hybrid generations can be reduced due to genetic incompatibilities between the parental genomes or hybrid breakdown of segregating hybrid offspring (Burke & Arnold, 2001). Further, disruption of resistance mechanisms can lead to higher susceptibility of hybrids to pathogens and herbivores (Fritz, 1999; Rieseberg & Carney, 1998). Thus, more parental‐like genotypes may demonstrate higher fitness than more intermediate genotypes (Burke & Arnold, 2001; Hallgren, Ikonen, Hjältén, & Roininen, 2003; Strauss, 1994). Arnold and Hodges (1995) reviewed several studies on hybrid fitness and found that hybrids showed a broad range of fitness values, which were also related to the class of the hybrids (F_1_, F_2_, backcrosses, etc.). In general, hybrid fertility increases after the F_1_ generation because endogenous and exogenous selection favor the fitter offspring (Arnold, 1997; Rieseberg & Carney, 1998). Thus, it is important to treat the different hybrid genotypes and generations separately in order to get an accurate estimate of hybrid fitness and to judge the evolutionary impact of hybridization correctly (Arnold & Hodges, 1995; Rieseberg, 1997).

Several models have been proposed that describe how hybrid zones are maintained, and try to predict the outcome of natural hybridization events. For the classification of hybrid zones, it is important to assess hybrid fitness in comparison with the fitness of the parental species and to determine whether selection for or against hybrids is endogenous or exogenous (Campbell & Waser, 2007; Hewitt, 1988). Three hybrid zone models, the Tension Zone model, the Mosaic model, and the Bounded Hybrid Superiority model, are mainly used to describe the structure of hybrid zones (Abbott & Brennan, 2014). Due to the presence of both parental and hybrid individuals in the study area, the Bounded Hybrid Superiority model can be excluded and thus we will focus on the Tension Zone and Mosaic model.

The Tension Zone model proposed by Key (1968) assumes that hybrids are consistently less fit than the parental species. Tension zones are therefore maintained by constant dispersal of purebred individuals into the hybrid zone and selection against hybrid genotypes (Barton & Hewitt, 1985). Selection against hybrids is endogenous, that is, only due to genetic incompatibilities of the parental genomes (Hewitt, 1988). Thus, tension zones are not bound to a certain environment (Key, 1968). If individuals of one parental species would spread into the range of the other parental species, they would mainly mate with the other species due to lack of conspecific mates and mainly form inviable hybrid offspring. Enclaves of one species surrounded by the other species should thus be eradicated (Key, 1968, 1981). Therefore, a tension zone, that is, the border area between two hybridizing species, should run as a line across the landscape (Key, 1968). Under the Tension Zone model, endogenous selection against hybrids prevents introgression so that the hybridizing species retain their purity (Hewitt, 1988).

The Mosaic model (Harrison, 1986; Howard, 1986) assumes that the hybridizing species are adapted to different environments that are patchily distributed across the landscape, intermingled with intermediate habitats enabling hybrid establishment. This patchy distribution of habitats in the Mosaic model contrasts the distribution of the species in the Tension Zone model. In the Mosaic model, hybridization between species will occur at the boundaries between the different habitat types (Harrison, 1990). The hybridization events in different patches are independent of each other and can lead to different outcomes depending on local conditions (Harrison & Rand, 1989). Thus, the Mosaic model includes both endogenous selection against hybrids and exogenous selection that favors hybrid genotypes (Abbott & Brennan, 2014). The coexistence of purebred species and fit hybrids in a mosaic hybrid zone can lead to introgression of genes from one species into the other (Arnold, 1997; Barton & Hewitt, 1985; Mallet, 2005). The mosaic structure, however, prevents the total extinction of either species and maintains diversity (Harrison, 1986, 1990). Hence, hybridization has different outcomes under the Tension Zone and the Mosaic model. The assignment to one of the models and the prediction of the long‐term effects of a hybridization event are especially interesting in recently established hybrid populations.

In this study, we examined the fitness of natural hybrids between *Salix purpurea* L. and *S. helvetica* Vill. that have recently emerged on alpine glacier forefields (Gramlich, Sagmeister, Dullinger, Hadacek, & Hörandl, 2016). *Salix purpurea* is a widespread lowland species associated with calcareous soils and balanced soil pH value, while *S. helvetica* is a subalpine to alpine species that prefers silicate bedrock with acidic soil (Hörandl, Florineth, & Hadacek, 2012; Schiechtl, 1992). In contrast to many other alpine willows, *S. helvetica* is a medium shrub of ca. (30) 50–80 (150) cm (Hörandl et al., 2012). *Salix purpurea* is a taller shrub that can reach up to 6 m in the lowland (Schiechtl, 1992), but on the glacier forefield, plants were just ca. 160–180 cm high (pers. obs. of the authors). Both species are diploid, and homoploid hybridization between these species has taken place due to secondary contact of the up‐moving colonizer *S. purpurea* with the native subalpine species *S. helvetica* on glacier forefields of the Swiss Alps (Gramlich et al., 2016). Population genetic analyses of those populations revealed that the mixed stands comprised the parental species, F_1_ hybrids, and later‐generation hybrids. An ecological analysis on niche differentiation showed that the hybrids occupied more extreme environmental niches than the purebred individuals (Gramlich et al., 2016). In this previous study, it was found that, among other factors, hybrids occurred on more acidic soil than either parental species. It was concluded that the hybrids could coexist with their parents due to the occupation of slightly different niches and that they thus have a certain potential for mid‐ to long‐term persistence in this location. We were therefore interested in the evolutionary consequences of this willow hybridization event. In the earlier study (Gramlich et al., 2016), the hybrid zones were tentatively assigned to the Mosaic model based on the spatial distribution of the hybrids and the parental species. Thus, additional data on fitness are needed to support this assignment.

We sampled catkins from naturally pollinated willow shrubs occurring in sympatry on a glacier forefield and measured capsule production, seed production, germination rate of seeds, and growth of seedlings (1) to assess the fitness of the hybrids in comparison with the fitness of the purebred species, (2) to test the hypothesis that later‐generation hybrids are fitter than F_1_ hybrids, (3) to test the hypothesis that hybrids performed better than the purebred species under acidic substrate conditions. Finally (4), we wanted to use the data on hybrid fitness and the geographical distribution patterns to assign the willow hybrid population to one of the hybrid zone models.

## 2 Methods

### 2.1 Sampling

**Figure 1:**
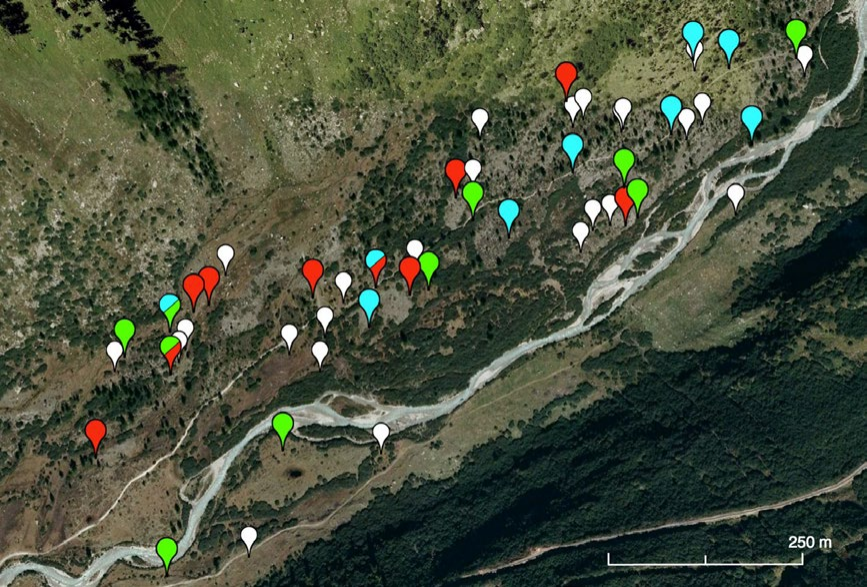
Distribution of the species on the forefield of the Rhône Glacier. The colored pins denote the locations of the individuals that were sampled for the fitness analysis in this study (red = *S. purpurea*, green = *S. helvetica*, blue = hybrid). The white pins denote the locations of individuals that were sampled in a previous study on population genetics (Gramlich et al., 2016). At each of these locations, *S. purpurea*,* S. helvetica,* and at least one hybrid occurred in close vicinity. (Map data: Google Earth, Flotron/Perrinjaquet.)

Catkins and seeds were sampled in the field from a mixed stand of *S. purpurea*,* S. helvetica,* and their hybrid, located at the forefield of the Rhône Glacier in central Switzerland (46°34′03.0″N, 08°22′12.3″E). This population has been the target of a previous study on population genetic structure. In this study, 182 individuals were genotyped using nine microsatellite loci and the genetic data were used to determine whether the sampled individuals had a purebred or hybrid genotype using Bayesian methods (NewHybrids analysis) (Gramlich et al., 2016). An individual was only assigned to a particular group (purebred, F_1_, F_2_, backcross to *S. purpurea* or *S. helvetica*) if the probability that this individual belonged to this group was ≥95%. Individuals that could not be unequivocally assigned to a group were regarded as later‐generation hybrids (F_2_ hybrids, backcrosses). On the basis of these results, we could sample catkins specifically from individuals that had already been genotyped and whose purebred or hybrid status was known. We focused on female fitness as male catkins fall off early, and it was difficult to collect sufficient materials in the natural population. Due to the low number of catkins on many known individuals, we had to sample eight additional plants that were genotyped and analyzed using the methods described in Gramlich et al. (2016). Altogether, thirty individuals were sampled, each ten of *S. purpurea*,* S. helvetica*, and hybrid individuals. One individual with a hybrid phenotype was assigned to *S. helvetica* in the genetic analysis and was removed from the fitness analyses due to this ambiguous assignment. Thus, the final sample size was 29 individuals. The locations of these individuals on the glacier forefield are shown in Figure 1.

### 2.2 Germination and growth experiments

To acquire seeds for the germination experiments, catkins with capsules that had just started to open were sampled and stored in paper bags overnight so that the capsules could fully open and release the seeds. Willow seeds are nondormant and short‐lived, as they are not tolerant to desiccation. For these reasons, rapid germination on a moist substrate is essential for reproductive success (Karrenberg & Suter, 2003). Thus, the seeds were used for the germination experiments within 24 h to ensure germination capacity. In order to test the hypothesis that hybrids could tolerate low pH values better than the purebred species, seeds were germinated in media with pH 4.5, pH 7, and pH 8, respectively. Buffered pH solutions were prepared with 2 mM MES (2‐(N‐morpholino)ethanesulfonic acid, Carl Roth, Karlsruhe, Germany) or 2 mM HEPES (4‐(2‐hydroxyethyl)‐1‐piperazineethanesulfonic acid, Carl Roth, Karlsruhe, Germany). The MES solution was titrated to pH 4.5 with 1 M HCl; the HEPES solution was titrated to pH 7 or pH 8 using 1 M NaOH (Reddy & Singh, 1992; Shaw, Mack, & Smith, 1991). The pH values of the solutions were measured with a pH meter (Hanna Instruments, Kehl, Germany). If possible, hundred seeds per plant and treatment were used in the germination experiment. However, due to low catkin production, only 6 to 90 seeds per treatment were available for ten individuals. The seeds were placed in 9‐cm petri dishes on filter paper which was wetted with the respective pH solution. The filter paper was partitioned in ten sections, and ten seeds of an individual were placed in each sector (Karrenberg & Suter, 2003). Seeds from the same plant were distributed over several petri dishes to minimize probable influences of the single petri dishes. For the duration of the germination experiment, the petri dishes were kept with closed lids in an unheated, well‐lit room (min. 12.5°C during a period of cold weather to max. 21°C on warm and sunny days) under daylight conditions of early to mid‐July (ca. 15.30 h daylight, similar to the natural conditions) near the Rhône Glacier. After one week, the germinated seeds were counted. Seeds were considered germinated when the cotyledons had appeared.

The seedlings were kept in the petri dishes for three weeks after the start of the germination experiment to monitor seedling development. The different pH treatments were continued, but after the first week, 1 ml of a 0.8 mg/L carbendazim solution (Sigma‐Aldrich, München, Germany) was added to 20 ml of pH solution to prevent mold formation on the filter paper. During the 3‐week period, the petri dishes were transferred to climate chambers at 18°C with a 16‐h light period (c. 250 μmol m^−2 ^s^−1^). After three weeks, height of the seedlings was measured.

### 2.3 Counting of capsules and seeds

Six to ten unripe catkins per plant were bagged in cellulose dialysis tubing (Visking, diameter 49 mm, Carl Roth, Karlsruhe, Germany) and sampled when the first capsules had started to open. The dialysis tubing prevented the loss of the seeds that had been released before the catkin was collected. We counted the total number of seeds per catkin and further discerned them in developed seeds (large, green, oval) and aborted seeds (small, yellow or brown, elongated). We also counted the number of capsules per catkin and discerned them in developed (ripe, open, releasing seeds, seed hairs inside) and unfertilized (unpollinated ovaries). These results were used to calculate the number of capsules per catkin, fruit set (percentage of developed fruits per catkin), seeds per developed capsule, and seed set (percentage of developed seeds among all seeds) as fitness parameters. Mean values for these parameters were calculated for each individual to avoid pseudoreplication.

### 2.4 Statistical analyses

The individuals were split into four groups for all statistical analyses. Two groups comprised the parental species *S. purpurea* (*n* = 10) and *S. helvetica* (*n* = 10). The hybrid individuals were divided into F_1_ hybrids (*n* = 5) and later‐generation hybrid classes (*n* = 4) based on the results of a previous population genetic study (Gramlich et al., 2016). Later‐generation hybrid classes comprise backcrosses to each parental species and probably F_2_ hybrids. After arcsine transformation of percentages, one‐way ANOVAs were performed to test for differences between groups, and Scheffé's test or the Games–Howell test were applied as post hoc tests. The type I error rate was α = 0.05. A power analysis was performed when the results were not statistically significant to account for the small and skewed sample size. All statistical analyses were performed using SPSS version 23 (IBM Corp., Armonk, NY, USA).

## 3 Results

### 3.1 Capsules and seeds

**Figure 2:**
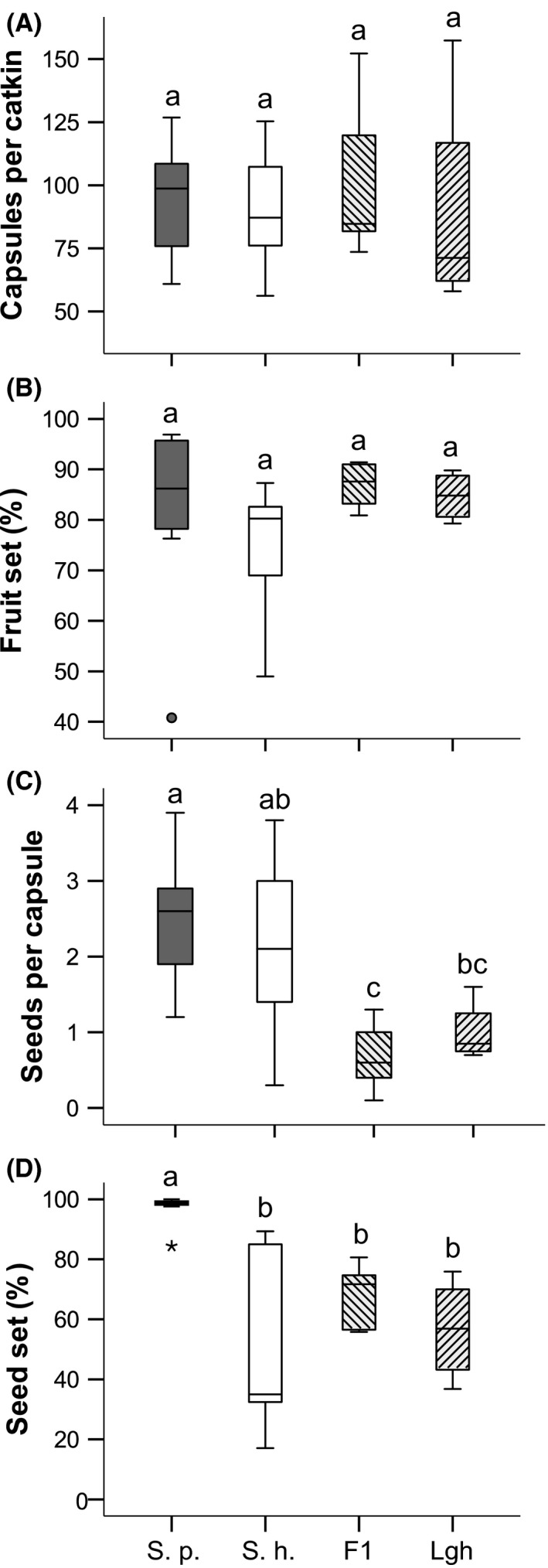
Comparison of fitness parameters among the four groups. The Box plots show (A) the number of capsules per catkin, (B) fruit set (percentage of developed fruits per catkin), (C) seeds per developed capsule, (D) percentage of developed seeds among all seeds produced by a single catkin. Significant differences are indicated by different letters. The bottom and top of the boxes show the 25 and 75 percentiles, respectively. The median is indicated by the black horizontal line. The whiskers extend to the highest and lowest value that is not an outlier. Outliers are indicated by circles, and stars indicate extreme values. (parents: S. p. = S. purpurea, S. h. = S. helvetica, F1 = F1 hybrids, Lgh = later‐generation hybrids)

The number of capsules per catkin did not differ significantly between the four groups (Fig. 2A, Table 1). Further, there were no significant differences in the percentage of developed fruits per catkin (Fig. 2B, Table 1). However, the power analysis showed that nonsignificant results can also be due to the low sample size and the lack of power (Table 1). Thus, all nonsignificant results should be interpreted with caution. However, the F_1_ hybrids produced significantly less seeds per capsule than both parental species (Table 2), and the later‐generation hybrids produced less seeds per capsule than *S. purpurea* (Fig. 2C, Table 2). Remarkably, the number of seeds per capsule was rather low even in the parental species. *Salix purpurea* has six ovules per fruit (Karrenberg & Suter, 2003), and thus, with 1–4 seeds per capsule, it becomes clear that not all ovaries have been fertilized (mean 41%). *Salix purpurea*, however, produced a significantly higher percentage of good, viable seeds than any other group, while there were no significant differences between *S. helvetica*, F_1_ hybrids, and later‐generation hybrids (Fig. 2D, Table 2).

**Table 1:** Differences between the four taxa (*S. purpurea*,* S. helvetica*, F_1_ hybrids, later‐generation hybrids) for various fitness parameters based on ANOVA. Results of the power analysis are given for nonsignificant results

Fitness parameter	*df*	MS	*F*‐value	*p*‐Value	1‐β
Germination rate pH 4.5	3, 25	0.146	6.042	.003	
Germination rate pH 7	3, 25	0.104	3.881	.021	
Germination rate pH 8	3, 25	0.061	2.718	.066	.1–.5
Plant height pH 4.5	3, 25	7.616	11.440	<.001	
Plant height pH 7	3, 25	8.369	11.725	<.001	
Plant height pH 8	3, 25	9.060	12.304	<.001	
Capsules per catkin	3, 25	196.308	0.260	.853	<.1
Fruit set	3, 25	0.037	1.565	.223	<.1
Seeds per capsule	3, 25	4.663	5.291	.006	
% developed seeds	3, 25	0.789	18.507	<.001	

**Table 2:** Significant differences between pairs of taxa (*p*‐values) for various fitness parameters as indicated by post hoc tests after ANOVA

	Comparison					
	*S. purpurea* vs. *S. helvetica*	*S. purpurea* vs. F_1_ hybrids	*S. purpurea* vs. Later‐generation hybrids	*S. helvetica* vs. F_1_ hybrids	*S. helvetica* vs. Later‐generation hybrids	*F*_1_ vs. Later‐generation hybrids
Germination pH 4.5^b^	.940	<.001	.142	.007	.576	.041
Germination pH 7^b^	.639	<.001	.113	.128	.808	.376
Plant height pH 4.5^a^	.001	.007	.003	.998	.969	.947
Plant height pH 7^a^	<.001	.005	.005	.999	1.000	.998
Plant height pH 8^a^	<.001	.001	.005	.983	.998	.999
Seeds per capsule^b^	.827	.001	.006	.038	.129	.709
% developed seeds^b^	<.001	<.001	.013	.391	.959	.702

Post hoc test: ^a^Scheffé test. ^b^Games–Howell test.

### 3.2 Germination rate

**Figure 3:**
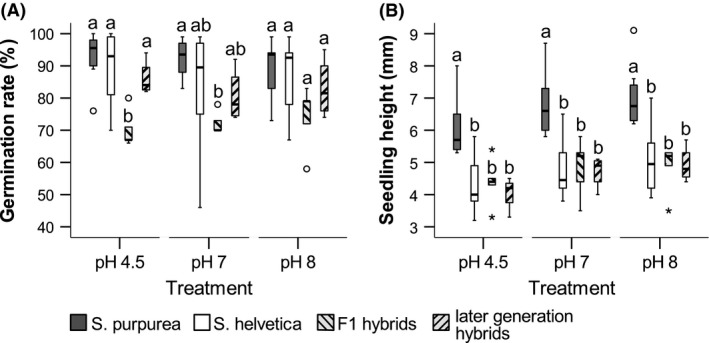
(A) Box plot of the effects of the pH treatment on germination rate measured after one week and (B) seedling height measured after 3 weeks. Letters indicate significant differences between groups within the respective treatment. It should be noticed that the shading of the bars indicates the genotype of the parent plant from which the seeds were collected and is therefore no indication of the genotype of the seeds or seedlings. The bottom and top of the boxes show the 25 and 75 percentiles, respectively. The median is indicated by the black horizontal line. The whiskers extend to the highest and lowest value that is not an outlier. Outliers are indicated by circles, and stars indicate extreme values

Generally, the germination rate was high across all species and treatments with median values exceeding 70% (Fig. 2A). The ANOVA revealed significant differences for the treatments with pH 4.5 and pH 7 (Table 1). Seeds collected from F_1_ hybrids showed the lowest mean germination rate in all pH treatments (Fig. 3A), but only in media with pH 4.5, they were significantly different from all other groups (Table 2). In the media with pH 7, the germination rate of seeds from F_1_ hybrids was only significantly lower than the germination rate of seeds from *S. purpurea*. Seeds from later‐generation hybrids did not differ in their germination rates from the parental species (Fig. 3A, Table 2). Further, there was no significant difference between the germination rate of seeds from *S. purpurea* and *S. helvetica*, irrespective of the treatment.

### 3.3 Growth and plant height

Seedlings of *S. purpurea* were significantly taller than the seedlings of any other group in all pH treatments, while there were no significant differences between seedlings of *S. helvetica*, F_1_ hybrids, and later‐generation hybrids (Fig. 3B, Table 1, Table 2). Interestingly, the hybrids did not show intermediate height between the parental species, but rather they seem to adopt the slower growth of *S. helvetica*. Overall, seedlings from hybrid mother plants developed as well as seedlings with purebred mothers and only one hybrid seedling (among >700 seedlings) showed abnormally shaped leaves.

## 4 Discussion

In this study, we present measures of female fitness of a recently emerged natural hybrid population on a glacier forefield, consisting of F_1_ and later‐generation hybrids (F_2_ and backcrosses). Such early generations can be rarely observed under natural conditions, but are important for the understanding of the evolutionary processes in hybrid zones (Arnold, 1997). Our model system represents a case of secondary contact hybridization after breakdown of ecological crossing barriers of the parents and is further facilitated by the presence of a pioneer habitat (see Gramlich et al., 2016).

### 4.1 Hybrid fitness

Hybrid fitness was assessed for the parameters fruit production, seed set, and germination rate. Hybrids of both classes demonstrated a lower fitness than the parental species with regard to seed production although having the same amount of capsules per catkin. These results are in accordance with a study on hybrids between *S. eriocephala* and *S. sericea* that revealed a reduced seed production in F_1_ and F_2_ hybrids (Fritz, Hochwender, Albrectsen, & Czesak, 2006). Apparently, even in *S. purpurea,* less than half of the available ovules have been fertilized so that plants of this species did not produce the theoretically possible number of seeds. However, a lowered number of fertilized ovules seems to be common in many *Salix* species and is probably due to pollen limitation (Elmqvist, Agren, & Tunlid, 1988; Karrenberg, Kollmann, & Edwards, 2002). Thus, the discrepancy between the high percentage of developed capsules and the low number of seeds per capsule in hybrid individuals may have additional causes. The abortion of seeds may be due to intrinsic selection against intermediate genotypes (Cruzan & Arnold, 1994). Possible prezygotic incompatibilities in *Salix* may comprise pollen‐pistil incongruities between species, resulting in failure of fertilization (Mosseler, 1989). Classical postzygotic mechanisms such as disturbance of meiosis and unequal segregation of chromosomes in the gametes, resulting in strongly reduced fertility of the adult F_1_ hybrids, probably act in willows as well (e.g., Mosseler, 1990). Further studies on the reproductive barriers are necessary to clarify the actual reasons for the low seed production of the willow hybrids. The greater reduction in seed production in the F_1_ hybrids compared with the later‐generation hybrids may be a hint that endogenous selection favors fitness in advanced hybrid generations. Positive selection for good seeds that have resulted from regular meiosis and gamete formation in the F_1_ will increase fertility in the subsequent generations (Grant, 1966). Further, the catkins of most hybrids were heavily infested with cicadas and rust fungi (pers. obs.) which may have damaged the capsules internally and decreased seed production. Similarly, Karrenberg and Suter (2003) observed a reduced fruit set in willows due to insect herbivory and fungal infestation. A higher susceptibility to fungi and insect pests can be caused by the breakup of resistance genes in hybrids (Fritz, Hochwender, Brunsfeld, & Roche, 2003; Rieseberg & Carney, 1998). In some willow species, it has also been observed that fruits developed occasionally although pollen had been excluded, yet these fruits did not produce seeds (Karrenberg, Kollmann et al., 2002). Although our data indicate that seed production per catkin is greatly reduced, the willow hybrids could still reach a relatively high seed set which is comparable to experimentally produced willow hybrids (Mosseler, 1990).

The field study was limited to female fitness, but at least, some of the hybrid seedlings of the germination experiment were raised to maturity in the botanical garden of the University of Göttingen for further examination of male fitness. Pollen staining of some of these hybrids (with 1% TTC solution) suggested that hybrids produce viable pollen, and catkins pollinated with hybrid pollen produced viable seeds (S. Gramlich, unpubl. data). Hence, male fitness is probably not a limiting factor for seed set. Unlike other alpine *Salix* species, *S. helvetica* does not show clonal growth, and population genetic data did not indicate any clonality in the willow populations on the glacier forefield (Gramlich et al., 2016). Hence, an influence of clonal reproduction on fitness was not relevant in this study, as it was the case in other studies on high alpine creeping *Salix* species (Sedlacek et al., 2016).

Individuals of the purebred species did not perform equally well in the fitness analyses. Individuals of *S. helvetica* often showed quite low values, while *S. purpurea* outperformed all other groups in some cases. The glacier forefield is situated at the lower boundary of the altitudinal range of *S. helvetica*. Thus, it may be possible that the conditions start to become unsuitable for this subalpine to alpine species and lead to a reduction in fertility (Lenoir, Gégout, Dupouey, Bert, & Svenning, 2010; Lesica & McCune, 2004). *Salix purpurea*, in contrast, is a widespread pioneer species with broad ecological amplitude (Hörandl et al., 2012; Schiechtl, 1992) so that fast growth and high seed production in *S. purpurea* are simply general features of a good colonizer.

The germination rate of seeds collected from hybrids and purebred individuals of each parental species is in the range of what is known from other willow hybrids (Mosseler, 1990). Germination was assessed in media with different pH values as another fitness parameter. The germination rate was quite high across all groups and treatments and exceeded 70% even in seeds collected from hybrid individuals. Nevertheless, seeds collected from F_1_ hybrids showed the lowest germination rate in all treatments while the germination rate of seeds collected from later‐generation hybrids was never significantly different from that of the purebred species. Hence, the germination ability seems to be restored in later hybrid generations by selection for good seed formation and is comparable to those of the parental species. Generally, freshly collected willow seeds exhibit high germination rates exceeding 80% (Karrenberg & Suter, 2003; van Splunder, Coops, Voesenek, & Blom, 1995), but lowered germination rates for hybrid seeds were also found in another study on willow hybrids (Fritz et al., 2006). Seeds of *S. purpurea* do not overwinter and lose their germination ability after about 2 weeks (Karrenberg & Suter, 2003). Some late‐flowering high alpine *Salix* species growing in snowbeds, on the other hand, delay germination to the next spring and need stratification to increase germination rate (Sedlacek, Bossdorf, Cortés, Wheeler, & van Kleunen, 2014). Seeds of *S. helvetica* germinated with the same rate as seeds of *S. purpurea* so that they do not seem to require cold treatment for germination. Due to this background, it seems unlikely that hybrid seeds would have needed stratification to increase germination rates. Overall, our findings suggest that hybrid seeds germinate well, at least under benign, artificial conditions.

Ecological data from the local habitat conditions of the adult plants suggested a preference of hybrids for acidic sites (Gramlich et al., 2016). Unexpectedly, the different pH treatments of seeds did not have much effect on the germination rate. Only in the substrate with pH 4.5, the germination rate of seeds collected from F_1_ hybrids was significantly lower than in any other group. Thus, we could not confirm our hypothesis that hybrid seedlings would perform better than the purebred species under acidic conditions. Further, there were no significant differences in the germination rates between *S. purpurea* and *S. helvetica* although *S. purpurea* is in general associated with calcareous soils while *S. helvetica* prefers silicate bedrock with slightly acidic soil pH (Hörandl et al., 2012; Schiechtl, 1992). Overall, the pH value does not seem to be crucial for the germination success of willow seeds and for early seedling development. These results are in accordance with other studies on willow seed germination that found that the most important factors for seed germination were constantly moist soil conditions (Castro‐Morales, Quintana‐Ascencio, Fauth, Ponzio, & Hall, 2014; McLeod & McPherson, 1973; van Splunder et al., 1995). For willow seedlings, light availability is essential, as seeds have no endosperm and seedling development depends completely on successful photosynthesis of the early leaves (Neumann, 1981). Further, seedlings of Salicaceae are shade‐intolerant and thus perform best on open areas without shading by taller vegetation (Karrenberg, Edwards, & Kollmann, 2002). Our previous study revealed that mature hybrid individuals prefer acidic soil conditions, while the results of the present study showed that the pH value of the growing medium does not have much effect on the germination rate of hybrid seeds. Thus, the requirements for the germination of seeds seem to be different from the requirements for the successful growth of seedlings so that the observed niche differences between the hybrids and the parental species seem to emerge in later stages of the life cycle. Soil pH strongly affects the uptake of nutrients and hence may influence the overall growth and vigor of the adult plants rather than the seedlings.

The measurement of seedling height three weeks after the start of the germination experiment showed that seedlings of *S. purpurea* were significantly taller than seedlings of any other group which is in line with the pioneer features of the plant (Hörandl et al., 2012). The plant height of both hybrid classes was not significantly different from seedlings of *S. helvetica*. Interestingly, the hybrids did not adopt the fast growth of *S. purpurea* and they were also not intermediate between the parents, as could be expected especially in the F_1_ hybrid generation. Rather, the hybrids adopted the slower growth of *S. helvetica*. In an alpine environment, a compact growth should be advantageous, especially in the early stages of the life cycle, because it enables the plant to stay below the snow cover during winter so that it will be better protected against frost and wind (Körner, 2003). On the glacier forefield, adult *S. purpurea* individuals were about 1.6–1.8 m high, while *S. helvetica* and hybrids reached ca. 0.5–0.8 m height (pers. obs. of authors). However, according to our vegetation data (Gramlich et al., 2016), the hybrid populations are still not dense enough so that no strong effects of facilitation/competition of neighboring individuals can be assumed. In our willow hybrids, slow growth may be associated with higher fitness so that the hybrids might be fitter than *S. purpurea* in the long term and equally fit as *S. helvetica*. Nagy (1997) studied the morphology of *Gilia* hybrids produced by species that were adapted to different environments and found that the morphology of the hybrids resembled that of the parent that was native to the respective sampling site. In accordance with this observation, the willow hybrid zone is situated in the subalpine zone that is the natural habitat of *S. helvetica* and the willow hybrids resemble the native species. Although seed production in the hybrids was reduced, the fitness of the hybrids in the seed‐to‐seedling stage seems to be comparable to the fitness of the purebred native species.

### 4.2 Hybrid zone models

Finally, we wanted to assign the willow hybrid zone to the Tension Zone model or the Mosaic model. In short, the Tension Zone model assumes that hybrids are consistently less fit than the parental species due to strong endogenous selection that prevents introgression between the parents (Barton & Hewitt, 1985; Hewitt, 1988; Key, 1968). The Mosaic model, on the other hand, assumes that the parental species and the hybrids are adapted to different environments that are patchily distributed across the landscape so that fit hybrids and their parents co‐occur, facilitating introgression between taxa (Barton & Hewitt, 1985; Harrison, 1986; Howard, 1986). Scale can be crucial for such an assignment because the outcome can differ between low and high resolution (Harrison & Rand, 1989). Thus, we consider the hybrid zone on the small scale of the glacier forefield and on the large scale across the Alps separately.

The glacier forefield (at 1,700–1,800 m ASL) is completely situated within the range of *S. helvetica*. Thus, it does not constitute an intermediate habitat or an environmental cline running down from the subalpine zone into the lowlands. Rather, it can be viewed as an abrupt discontinuity in the landscape, as a local pioneer habitat that has become available due to the rapid retreat of glaciers. In this subalpine zone, the different willow species on the glacier forefield are forced to flower at the same time in the short summer vegetation period (June–July; pers. obs. by the authors). The onset of flowering in alpine habitats is strongly influenced by the timing of snowmelt as has been shown for willows and other species (Molau, Nordenhäll, & Eriksen, 2005; Sedlacek et al., 2015; Wheeler et al., 2016). In the alpine zone, there is a strong small‐scale differentiation in snow‐melt timing (Cortés et al., 2014; Wheeler et al., 2014) but due to the lower location in the subalpine zone (at 1,700–1,800 m ASL) and the flat surface topography of the glacier forefield in this study, the differences between microhabitats should be less pronounced. Hence, the difference in phenology between the lowland species *S. purpurea* and the alpine species *S. helvetica*, which is otherwise an important prezygotic reproductive barrier in willows (Mosseler & Papadopol, 1989), disappeared on the glacier forefield. However, this restricted, still unforested area seems to enable the overlap of the parental species’ ranges and the establishment of their hybrids. Further, there is no spatial cline from one species to the other as postulated by the Tension Zone model. Hybrid individuals with various phenotypes and genotypes and individuals of both purebred parental species are evenly dispersed across the glacier forefield with only a few meters distance between the single individuals (Gramlich et al., 2016), resembling a mosaic (Fig. 1). With regard to hybrid fitness, our analyses indicate that while fruit production is normal, seed production is significantly reduced, most likely due to endogenous selection during gamete formation, fertilization, or seed development. Other fitness parameters were, however, comparable to one or both parental species. Further, a previous genetic analysis of this hybrid zone revealed that the hybrid population was largely composed of later‐generation hybrids (Gramlich et al., 2016). Thus, endogenous selection against hybrids seems to be relatively weak in later stages of the life cycle, because otherwise the population should mainly consist of F_1_ hybrids (Baker, Davis, Bradley, Hamilton, & van Den Bussche, 1989; van den Bussche et al., 1993). Instead, endogenous selection appears to restore seed set and germination ability over generations. Thus, the strong endogenous selection proposed by the Tension Zone model is missing. Exogenous selection against hybrids on the glacier forefield does not seem to be strong either. This is probably due to the large number of unoccupied niches on the glacier forefield that allow for coexistence of the parental species and the hybrids (Gramlich et al., 2016). Moisture and light, as most important parameters for the establishment of willow seedlings, are obviously sufficiently available in the loose pioneer vegetation of the glacier moraines. There is probably a strong exogenous selection against hybrids outside the glacier forefield in the surrounding dense subalpine shrub and grassland vegetation, but this could only be confirmed by additional studies. In summary, it can be said that the Mosaic model best describes the willow hybrid zone on this glacier forefield. Some other studies have described hybrid zones in comparable detail, but unfortunately, they did not assign them to one of the current hybrid zone models (Cerón‐Souza et al., 2014; Field, Ayre, Whelan, & Young, 2011; Muranishi, Tamaki, Setsuko, & Tomaru, 2013; Valbuena‐Carabaña et al., 2005). Two studies, however, also revealed that hybridization and hybrid establishment were associated with habitat disturbance and assigned these zones to the Mosaic model (Lexer, Fay, Joseph, Nica, & Heinze, 2005; Milne & Abbott, 2008). The assignment does of course reflect the current situation on the glacier forefield and may change with natural succession of vegetation, progressing climate change, and ongoing colonization and establishment of further willow generations (Whittaker, 1993). Hence, we regard this hybrid zone as a special case and call it a “pioneer mosaic hybrid zone”.

It seems also to be important to assign the willow hybrid zone on a large scale. Many studies investigated hybridization in large zones of range overlap between species. Populations were sampled along transects spanning over 100 km up to 1,000 km. The hybridizing species showed a differentiation by altitude and/or environmental gradients in most cases (Cullingham, James, Cooke, & Coltman, 2012; Hersch‐Green, Allan, & Whitham, 2014; Howard, Preszler, Williams, Fenchel, & Boecklen, 1997; James & Abbott, 2005; Lexer et al., 2005; Peñaloza‐Ramírez et al., 2010; Raudnitschka, Hensen, & Oberprieler, 2007). If purebred and mixed populations were patchily distributed, so that there was no gradual change from one species to the other along the transect, the hybrid zone was assigned to the Mosaic model (Cullingham et al., 2012; Howard et al., 1997; Lexer et al., 2005; Ortego, Gugger, Riordan, & Sork, 2014; Peñaloza‐Ramírez et al., 2010; Raudnitschka et al., 2007). *Salix purpurea* and *S. helvetica* show differentiation by altitude in their distribution. Although *S. purpurea* occurs in lowlands or lower altitudes along the valleys, *S. helvetica* occurs in the subalpine zone. In aerial view, this pattern resembles a mosaic consisting of patches of *S. helvetica* restricted to sites in higher altitudes, patches of *S. purpurea* along the adjacent valleys, and a few sites of range overlap between species where the hybrid zones occur (see Appendix 1 for illustration). Thus, if a transect would be laid across the Alps and populations were sampled in regular distances, populations of the purebred species and hybrid populations would occur alternately. The spatial distribution of the hybrid zones also argues for the Mosaic model. Besides the hybrid population on the Rhône Glacier, we found another hybrid zone that established independently on the forefield of the Morteratsch Glacier. This second hybrid zone was not used for the fitness study due to the low number of hybrids, and the younger successive stage of this site (Gramlich et al., 2016). Nevertheless, independent local contacts of the hybridizing species are another key feature of mosaic hybrid zones (Harrison, 1990; Harrison & Rand, 1989). Thus, we would argue that on a large scale, the hybrid zone between *S. purpurea* and *S. helvetica* also resembles a pioneer mosaic hybrid zone.

Overall, although hybrids showed a reduced seed output, the seeds they produced demonstrated high germination ability and hybrid seedlings developed as well as purebred ones. The specific circumstances on the glacier forefield seem to weaken exogenous selection against hybrid genotypes. Thus, hybrids have the potential to persist, at least as long as the glacier forefield is in an early successional stage. While the parental species and the hybrids coexist, hybridization between *S. purpurea* and *S. helvetica* could lead to interspecific gene flow and introgression in this mosaic hybrid zone.

## Funding Information

Ursula Hofmann Stiftung of the University of Göttingen, and Deutsche Forschungsgemeinschaft (Grant/Award Number: “DFG project Ho 5462/7‐1”).

## Conflict of Interest

None declared.

## Appendix 1

### 1.1

Illustration of the mosaic‐like pattern of distribution of *Salix purpurea* and *S. helvetica* on a larger scale. Although *S. helvetica* is restricted to higher altitudes (green shading), *S. purpurea* occurs along the valleys at low altitudes (red shading), so that their ranges do not overlap. Hybrid zones (yellow) occur where the two species come into secondary contact. In aerial view, this pattern resembles a mosaic. Please note that the illustration does not reflect any real distribution of the species. (Image source: geodata © swisstopo).
